# 3-[4-(Benz­yloxy)phen­yl]-1-(2-fur­yl)-3-hydroxy­prop-2-en-1-one

**DOI:** 10.1107/S1600536808036659

**Published:** 2008-11-13

**Authors:** Chun-Yang Zheng, Dun-Jia Wang, Ling Fan

**Affiliations:** a Hubei Key Laboratory of Bioanalytical Techniques, Hubei Normal University, Huangshi 435002, People’s Republic of China; b College of Chemistry and Environmental Engineering, Hubei Normal University, Huangshi 435002, People’s Republic of China

## Abstract

In the crystal structure of the title compound, C_20_H_16_O_4_, which is in the enol form, the central benzene ring makes dihedral angles of 63.42 (9) and 5.19 (10)° with the phenyl and furan rings, respectively. There is a short strong intra­molecular O—H⋯O hydrogen bond.

## Related literature

For hydrogen bonds in 1,3-diketones, see: Bertolasi *et al.* (1991 ▶); Gilli *et al.* (2004 ▶); Vila *et al.* (1991 ▶). For 1,3-diketones as ligands, see: Baskar & Roesky (2005 ▶); Bassett *et al.* (2004 ▶); Jang *et al.* (2006 ▶); Soldatov *et al.* (2003 ▶).
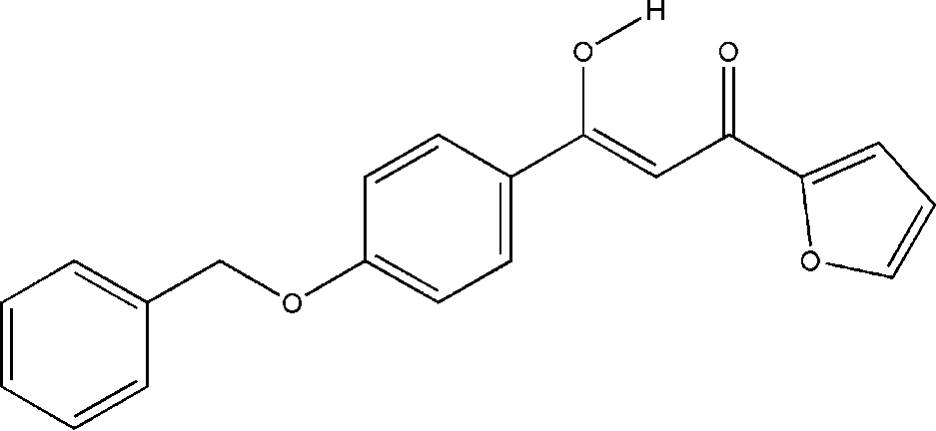


## Experimental

### 

#### Crystal data

C_20_H_16_O_4_
*M* *_r_* = 320.33
Triclinic, 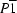

*a* = 5.8927 (6) Å
*b* = 11.3365 (11) Å
*c* = 13.3039 (13) Å
α = 112.111 (3)°
β = 96.687 (3)°
γ = 98.638 (3)°
*V* = 799.39 (14) Å^3^
*Z* = 2
Mo *K*α radiation
μ = 0.09 mm^−1^
*T* = 298 (2) K
0.32 × 0.20 × 0.12 mm

#### Data collection

Bruker SMART APEX CCD area-detector diffractometer
Absorption correction: multi-scan (**SADABS**; Sheldrick, 1996 ▶) *T*_min_ = 0.978, *T*_max_ = 0.983
6611 measured reflections
3439 independent reflections
2268 reflections with *I* > 2σ(*I*)
*R*_int_ = 0.078

#### Refinement

*R*[*F* ^2^ > 2σ(*F* ^2^)] = 0.056
*wR*(*F* ^2^) = 0.147
*S* = 0.95
3439 reflections
220 parameters
H atoms treated by a mixture of independent and constrained refinement
Δρ_max_ = 0.18 e Å^−3^
Δρ_min_ = −0.29 e Å^−3^

### 

Data collection: *SMART* (Bruker, 1997 ▶); cell refinement: *SAINT* (Bruker, 1999 ▶); data reduction: *SAINT*; program(s) used to solve structure: *SHELXS97* (Sheldrick, 2008 ▶); program(s) used to refine structure: *SHELXL97* (Sheldrick, 2008 ▶); molecular graphics: *SHELXTL* (Sheldrick, 2008 ▶); software used to prepare material for publication: *SHELXTL*.

## Supplementary Material

Crystal structure: contains datablocks global, I. DOI: 10.1107/S1600536808036659/is2357sup1.cif

Structure factors: contains datablocks I. DOI: 10.1107/S1600536808036659/is2357Isup2.hkl

Additional supplementary materials:  crystallographic information; 3D view; checkCIF report

## Figures and Tables

**Table 1:** Hydrogen-bond geometry (Å, °)

*D*—H⋯*A*	*D*—H	H⋯*A*	*D*⋯*A*	*D*—H⋯*A*
O2—H2*A*⋯O3	1.15 (3)	1.38 (3)	2.5030 (16)	162 (2)

**Fig. 1.**
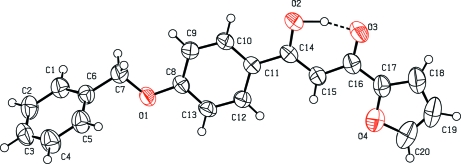
View of (I), showing the atom-labeling scheme. Displacement ellipsoids are drawn at the 50% probability level. The dashed line indicates an intramolecular hydrogen bond.

## supplementary crystallographic information

### Comment

1,3-Diketones in their enolic tautomeric forms have been extensively studied owing to their ability to form strong intermolecular or intramolecular hydrogen bonds (Vila *et al.*, 1991; Bertolasi *et al.*, 1991; Gilli *et al.*, 2004). They are among the most studied ligands in the chemistry of metal complexes and used widely in the chemistry of metallocomplexes (Baskar & Roesky, 2005; Bassett *et al.*, 2004; Jang *et al.*, 2006; Soldatov *et al.*, 2003).

The crystal structure of the title compound, (I), is in the enol form stabilized by an intramolecular hydrogen bond (Fig. 1). The distances of O2—H2 and O3···H2 are 1.15 (3) and 1.38 (3) Å, respectively. The central benzene ring (C8—C13) makes dihedral angles of 63.42 and 5.19° with two aromatic rings (C1—C6) and (C17—O4), respectively. The crystal packing is stabilized by van der Waals forces.

### Experimental

1-[4-(Benzyloxy)phenyl]ethanone (2.26 g, 0.01 mol), methyl furan-2-carboxylate (1.26 g, 0.01 mol), NaNH_2_ (0.78 g, 0.02 mol) and dry ether (60 ml) were placed into round bottom flask. The mixture was stirred for 6 h at room temperature under a blanket of nitrogen, acidified with dilute hydrochloric acid, and stirring was continued until all solids dissolved. The ether layer was separated and washed with saturated NaHCO_3_ solution, dried over anhydrous Na_2_SO_4_ and was removed by evaporation. The residual solid was recrystallized from an ethanol solution to give the title compound (I) (yield 1.75 g, 54.7%; m.p. 403 K). Crystals suitable for X-ray diffraction were grown by slow evaporation of a CH_2_Cl_2_—EtOH (1:4) solution at room temperature.

### Refinement

H atoms were placed in geometrically idealized positions and constrained to ride on their parent atoms, with C—H distances of 0.93 to 0.97 Å, and with *U*_iso_(H) = 1.2*U*_eq_(C). The H atom of the hydroxyl group was located in a difference Fourier map and its position was refined freely, with *U*_iso_(H) = 1.5*U*_eq_(O).

### Crystal data

**Table:**

C_20_H_16_O_4_	*Z* = 2
*M**_r_* = 320.33	*F*_000_ = 336
Triclinic, *P*1	*D*_x_ = 1.331 Mg m^−^^3^
Hall symbol: -P 1	Melting point: 403 K
*a* = 5.8927 (6) Å	Mo *K*α radiation λ = 0.71073 Å
*b* = 11.3365 (11) Å	Cell parameters from 1997 reflections
*c* = 13.3039 (13) Å	θ = 3.1–26.1º
α = 112.111 (3)º	µ = 0.09 mm^−^^1^
β = 96.687 (3)º	*T* = 298 (2) K
γ = 98.638 (3)º	Block, yellow
*V* = 799.39 (14) Å^3^	0.32 × 0.20 × 0.12 mm

### Data collection

**Table:**

Bruker SMART APEX CCD area-detector diffractometer	3439 independent reflections
Radiation source: fine-focus sealed tube	2268 reflections with *I* > 2σ(*I*)
Monochromator: graphite	*R*_int_ = 0.078
*T* = 298(2) K	θ_max_ = 27.0º
φ and ω scans	θ_min_ = 1.7º
Absorption correction: multi-scan(*SADABS*; Sheldrick, 1996)	*h* = −7→7
*T*_min_ = 0.978, *T*_max_ = 0.983	*k* = −14→14
6611 measured reflections	*l* = −16→16

### Refinement

**Table:**

Refinement on *F*^2^	Secondary atom site location: difference Fourier map
Least-squares matrix: full	Hydrogen site location: inferred from neighbouring sites
*R*[*F*^2^ > 2σ(*F*^2^)] = 0.056	H atoms treated by a mixture of independent and constrained refinement
*wR*(*F*^2^) = 0.147	*w* = 1/[σ^2^(*F*_o_^2^) + (0.0676*P*)^2^] where *P* = (*F*_o_^2^ + 2*F*_c_^2^)/3
*S* = 0.95	(Δ/σ)_max_ < 0.001
3439 reflections	Δρ_max_ = 0.18 e Å^−^^3^
220 parameters	Δρ_min_ = −0.29 e Å^−^^3^
Primary atom site location: structure-invariant direct methods	Extinction correction: none

### Special details

**Table:**

Geometry. All e.s.d.'s (except the e.s.d. in the dihedral angle between two l.s. planes) are estimated using the full covariance matrix. The cell e.s.d.'s are taken into account individually in the estimation of e.s.d.'s in distances, angles and torsion angles; correlations between e.s.d.'s in cell parameters are only used when they are defined by crystal symmetry. An approximate (isotropic) treatment of cell e.s.d.'s is used for estimating e.s.d.'s involving l.s. planes.
Refinement. Refinement of *F*^2^ against ALL reflections. The weighted *R*-factor *wR* and goodness of fit *S* are based on *F*^2^, conventional *R*-factors *R* are based on *F*, with *F* set to zero for negative *F*^2^. The threshold expression of *F*^2^ > σ(*F*^2^) is used only for calculating *R*-factors(gt) *etc*. and is not relevant to the choice of reflections for refinement. *R*-factors based on *F*^2^ are statistically about twice as large as those based on *F*, and *R*- factors based on ALL data will be even larger.

### Fractional atomic coordinates and isotropic or equivalent isotropic displacement parameters (Å^2^)

**Table:**

	*x*	*y*	*z*	*U*_iso_*/*U*_eq_	
C1	−0.1919 (3)	0.94140 (17)	−0.21175 (14)	0.0561 (4)	
H1	−0.2852	0.9490	−0.1588	0.067*	
C2	−0.2151 (3)	1.01001 (18)	−0.27760 (15)	0.0624 (5)	
H2	−0.3229	1.0636	−0.2686	0.075*	
C3	−0.0791 (3)	0.99904 (18)	−0.35624 (13)	0.0628 (5)	
H3	−0.0943	1.0449	−0.4008	0.075*	
C4	0.0791 (4)	0.9200 (2)	−0.36863 (14)	0.0707 (5)	
H4	0.1716	0.9124	−0.4219	0.085*	
C5	0.1021 (3)	0.85205 (18)	−0.30303 (14)	0.0625 (5)	
H5	0.2100	0.7986	−0.3124	0.075*	
C6	−0.0332 (3)	0.86224 (15)	−0.22321 (12)	0.0470 (4)	
C7	−0.0042 (3)	0.78860 (17)	−0.15223 (14)	0.0543 (4)	
H7A	−0.1266	0.7946	−0.1088	0.065*	
H7B	−0.0133	0.6975	−0.1976	0.065*	
C8	0.2985 (3)	0.78337 (14)	−0.01798 (12)	0.0437 (4)	
C9	0.1650 (3)	0.67956 (17)	−0.00705 (15)	0.0572 (5)	
H9	0.0099	0.6479	−0.0432	0.069*	
C10	0.2624 (3)	0.62314 (17)	0.05756 (14)	0.0566 (5)	
H10	0.1708	0.5530	0.0641	0.068*	
C11	0.4923 (3)	0.66708 (14)	0.11326 (12)	0.0430 (4)	
C12	0.6215 (3)	0.77425 (15)	0.10371 (12)	0.0487 (4)	
H12	0.7755	0.8073	0.1413	0.058*	
C13	0.5265 (3)	0.83230 (15)	0.04000 (13)	0.0493 (4)	
H13	0.6155	0.9047	0.0357	0.059*	
C14	0.5877 (3)	0.60034 (15)	0.17882 (12)	0.0459 (4)	
C15	0.8145 (3)	0.63971 (16)	0.23933 (13)	0.0493 (4)	
H15	0.9149	0.7099	0.2370	0.059*	
C16	0.8939 (3)	0.57587 (17)	0.30329 (13)	0.0523 (4)	
C17	1.1263 (3)	0.62181 (18)	0.37217 (13)	0.0561 (4)	
C18	1.2389 (4)	0.5838 (2)	0.44496 (16)	0.0779 (6)	
H18	1.1821	0.5149	0.4632	0.094*	
C19	1.4595 (4)	0.6689 (3)	0.48793 (17)	0.0878 (7)	
H19	1.5767	0.6671	0.5402	0.105*	
C20	1.4681 (4)	0.7517 (2)	0.43989 (17)	0.0821 (7)	
H20	1.5957	0.8185	0.4537	0.099*	
O1	0.22079 (19)	0.84506 (10)	−0.08083 (9)	0.0549 (3)	
O2	0.4480 (2)	0.49997 (11)	0.17879 (10)	0.0593 (3)	
O3	0.7661 (2)	0.47547 (13)	0.30691 (11)	0.0682 (4)	
O4	1.2673 (2)	0.72692 (13)	0.36792 (9)	0.0661 (4)	
H2A	0.573 (4)	0.473 (2)	0.2360 (18)	0.099*	

### Atomic displacement parameters (Å^2^)

**Table:**

	*U*^11^	*U*^22^	*U*^33^	*U*^12^	*U*^13^	*U*^23^
C1	0.0612 (10)	0.0584 (11)	0.0545 (10)	0.0197 (8)	0.0132 (8)	0.0256 (9)
C2	0.0691 (11)	0.0551 (11)	0.0649 (11)	0.0249 (9)	0.0015 (9)	0.0249 (9)
C3	0.0835 (13)	0.0586 (11)	0.0460 (10)	0.0101 (10)	−0.0026 (9)	0.0268 (9)
C4	0.0881 (13)	0.0858 (15)	0.0494 (10)	0.0291 (11)	0.0207 (10)	0.0330 (10)
C5	0.0706 (11)	0.0704 (12)	0.0559 (10)	0.0315 (9)	0.0166 (9)	0.0281 (9)
C6	0.0533 (9)	0.0446 (9)	0.0408 (8)	0.0094 (7)	0.0023 (7)	0.0167 (7)
C7	0.0536 (9)	0.0518 (10)	0.0594 (10)	0.0085 (7)	0.0032 (8)	0.0277 (8)
C8	0.0538 (9)	0.0380 (8)	0.0411 (8)	0.0099 (7)	0.0076 (7)	0.0181 (7)
C9	0.0489 (9)	0.0528 (10)	0.0709 (11)	−0.0024 (7)	−0.0039 (8)	0.0354 (9)
C10	0.0540 (10)	0.0505 (10)	0.0702 (11)	−0.0013 (8)	0.0000 (8)	0.0375 (9)
C11	0.0491 (8)	0.0407 (8)	0.0413 (8)	0.0096 (7)	0.0095 (7)	0.0183 (7)
C12	0.0481 (9)	0.0494 (9)	0.0481 (9)	0.0015 (7)	0.0031 (7)	0.0239 (8)
C13	0.0536 (9)	0.0434 (9)	0.0515 (9)	−0.0011 (7)	0.0056 (7)	0.0251 (8)
C14	0.0568 (9)	0.0432 (9)	0.0421 (8)	0.0121 (7)	0.0144 (7)	0.0198 (7)
C15	0.0556 (9)	0.0494 (9)	0.0468 (9)	0.0114 (7)	0.0077 (7)	0.0239 (8)
C16	0.0633 (10)	0.0554 (11)	0.0463 (9)	0.0246 (8)	0.0171 (8)	0.0230 (8)
C17	0.0650 (10)	0.0659 (12)	0.0482 (10)	0.0312 (9)	0.0164 (8)	0.0267 (9)
C18	0.0854 (15)	0.1060 (17)	0.0697 (12)	0.0522 (13)	0.0222 (11)	0.0510 (12)
C19	0.0787 (15)	0.128 (2)	0.0584 (13)	0.0552 (14)	0.0019 (10)	0.0304 (13)
C20	0.0648 (12)	0.0965 (17)	0.0662 (13)	0.0287 (11)	−0.0040 (10)	0.0124 (12)
O1	0.0627 (7)	0.0467 (7)	0.0560 (7)	0.0009 (5)	−0.0054 (5)	0.0302 (6)
O2	0.0605 (7)	0.0568 (7)	0.0712 (8)	0.0060 (6)	0.0086 (6)	0.0408 (7)
O3	0.0772 (9)	0.0687 (9)	0.0799 (9)	0.0223 (7)	0.0166 (7)	0.0498 (7)
O4	0.0671 (8)	0.0683 (9)	0.0576 (8)	0.0189 (7)	0.0014 (6)	0.0208 (7)

### Geometric parameters (Å, °)

**Table:**

C1—C6	1.373 (2)	C10—H10	0.9300
C1—C2	1.381 (2)	C11—C12	1.390 (2)
C1—H1	0.9300	C11—C14	1.469 (2)
C2—C3	1.371 (3)	C12—C13	1.373 (2)
C2—H2	0.9300	C12—H12	0.9300
C3—C4	1.369 (3)	C13—H13	0.9300
C3—H3	0.9300	C14—O2	1.3000 (19)
C4—C5	1.373 (2)	C14—C15	1.391 (2)
C4—H4	0.9300	C15—C16	1.393 (2)
C5—C6	1.382 (2)	C15—H15	0.9300
C5—H5	0.9300	C16—O3	1.287 (2)
C6—C7	1.489 (2)	C16—C17	1.456 (3)
C7—O1	1.4364 (19)	C17—C18	1.347 (2)
C7—H7A	0.9700	C17—O4	1.371 (2)
C7—H7B	0.9700	C18—C19	1.408 (3)
C8—O1	1.3583 (17)	C18—H18	0.9300
C8—C9	1.379 (2)	C19—C20	1.318 (3)
C8—C13	1.386 (2)	C19—H19	0.9300
C9—C10	1.374 (2)	C20—O4	1.352 (2)
C9—H9	0.9300	C20—H20	0.9300
C10—C11	1.385 (2)	O2—H2A	1.15 (3)
			
C6—C1—C2	121.06 (16)	C10—C11—C14	119.56 (13)
C6—C1—H1	119.5	C12—C11—C14	123.34 (14)
C2—C1—H1	119.5	C13—C12—C11	121.39 (14)
C3—C2—C1	119.91 (17)	C13—C12—H12	119.3
C3—C2—H2	120.0	C11—C12—H12	119.3
C1—C2—H2	120.0	C12—C13—C8	120.24 (14)
C4—C3—C2	119.52 (16)	C12—C13—H13	119.9
C4—C3—H3	120.2	C8—C13—H13	119.9
C2—C3—H3	120.2	O2—C14—C15	119.95 (14)
C3—C4—C5	120.50 (17)	O2—C14—C11	116.68 (14)
C3—C4—H4	119.8	C15—C14—C11	123.37 (14)
C5—C4—H4	119.8	C14—C15—C16	121.14 (15)
C4—C5—C6	120.71 (17)	C14—C15—H15	119.4
C4—C5—H5	119.6	C16—C15—H15	119.4
C6—C5—H5	119.6	O3—C16—C15	122.40 (16)
C1—C6—C5	118.30 (15)	O3—C16—C17	116.31 (15)
C1—C6—C7	121.80 (15)	C15—C16—C17	121.28 (16)
C5—C6—C7	119.90 (15)	C18—C17—O4	109.43 (17)
O1—C7—C6	107.58 (12)	C18—C17—C16	133.19 (19)
O1—C7—H7A	110.2	O4—C17—C16	117.37 (14)
C6—C7—H7A	110.2	C17—C18—C19	106.5 (2)
O1—C7—H7B	110.2	C17—C18—H18	126.7
C6—C7—H7B	110.2	C19—C18—H18	126.7
H7A—C7—H7B	108.5	C20—C19—C18	106.89 (19)
O1—C8—C9	124.48 (14)	C20—C19—H19	126.6
O1—C8—C13	116.21 (13)	C18—C19—H19	126.6
C9—C8—C13	119.29 (14)	C19—C20—O4	111.2 (2)
C10—C9—C8	119.65 (15)	C19—C20—H20	124.4
C10—C9—H9	120.2	O4—C20—H20	124.4
C8—C9—H9	120.2	C8—O1—C7	118.05 (11)
C9—C10—C11	122.26 (15)	C14—O2—H2A	99.0 (11)
C9—C10—H10	118.9	C16—O3—H2A	95.5 (9)
C11—C10—H10	118.9	C20—O4—C17	105.94 (16)
C10—C11—C12	117.10 (14)		
			
C6—C1—C2—C3	0.3 (3)	C12—C11—C14—O2	−178.85 (14)
C1—C2—C3—C4	−0.1 (3)	C10—C11—C14—C15	−178.45 (15)
C2—C3—C4—C5	0.0 (3)	C12—C11—C14—C15	1.4 (2)
C3—C4—C5—C6	−0.1 (3)	O2—C14—C15—C16	−2.1 (2)
C2—C1—C6—C5	−0.4 (2)	C11—C14—C15—C16	177.71 (14)
C2—C1—C6—C7	179.38 (15)	C14—C15—C16—O3	2.4 (2)
C4—C5—C6—C1	0.4 (3)	C14—C15—C16—C17	−176.10 (14)
C4—C5—C6—C7	−179.47 (17)	O3—C16—C17—C18	−3.0 (3)
C1—C6—C7—O1	−111.01 (17)	C15—C16—C17—C18	175.63 (18)
C5—C6—C7—O1	68.82 (19)	O3—C16—C17—O4	178.65 (13)
O1—C8—C9—C10	179.06 (15)	C15—C16—C17—O4	−2.7 (2)
C13—C8—C9—C10	−2.5 (3)	O4—C17—C18—C19	0.0 (2)
C8—C9—C10—C11	0.2 (3)	C16—C17—C18—C19	−178.42 (17)
C9—C10—C11—C12	1.7 (3)	C17—C18—C19—C20	0.0 (2)
C9—C10—C11—C14	−178.47 (15)	C18—C19—C20—O4	0.0 (2)
C10—C11—C12—C13	−1.3 (2)	C9—C8—O1—C7	−8.7 (2)
C14—C11—C12—C13	178.88 (14)	C13—C8—O1—C7	172.83 (13)
C11—C12—C13—C8	−1.0 (2)	C6—C7—O1—C8	−171.88 (13)
O1—C8—C13—C12	−178.53 (13)	C19—C20—O4—C17	0.0 (2)
C9—C8—C13—C12	2.9 (2)	C18—C17—O4—C20	−0.02 (19)
C10—C11—C14—O2	1.3 (2)	C16—C17—O4—C20	178.72 (15)

### Hydrogen-bond geometry (Å, °)

**Table:**

*D*—H···*A*	*D*—H	H···*A*	*D*···*A*	*D*—H···*A*
O2—H2A···O3	1.15 (3)	1.38 (3)	2.5030 (16)	162 (2)
